# No increase in postoperative contacts with the healthcare system following outpatient total hip and knee arthroplasty

**DOI:** 10.1080/17453674.2021.1922966

**Published:** 2021-05-12

**Authors:** Christian E Husted, Henrik Husted, Christian Skovgaard Nielsen, Mette Mikkelsen, Anders Troelsen, Kirill Gromov

**Affiliations:** Department of Orthopedic Surgery, Copenhagen University Hospital, Hvidovre, Denmark

## Abstract

Background and purpose — Discharge on the day of surgery (DDOS) after total hip arthroplasty (THA) and total knee arthroplasty (TKA) has been shown to be safe in selected patients. Concerns have been raised that discharging patients on the day of surgery (DOS) could lead to an increased burden on other parts of the healthcare system when compared with patients not discharged on the DOS (nDDOS). Therefore, we investigated whether discharging patients on the day of surgery (DOS) after THA and TKA leads to increased contacts with the primary care sector or other departments within the secondary care sector.

Patients and methods — Prospective data on 261 consecutive patients scheduled for outpatient THA (n = 135) and TKA (n = 126) were collected as part of a previous cohort study. 33% of THA patients and 37% of TKA patients were discharged on the DOS. Readmissions within 3 months after surgery were recorded. Contacts with the discharging department, other departments, and primary care physicians within 3 weeks were registered.

Results — No statistically significant differences were found when comparing DDOS patients and patients not discharged on the DOS (nDDOS) with regard to readmissions, physical contacts with the discharging department, and contacts with other departments as well as general practitioners. THA DDOS patients had significantly fewer contacts with the discharging department by telephone than THA nDDOS patients. TKA DDOS patients had significantly more contacts with the discharging department by telephone than TKA nDDOS patients.

Interpretation — Patients discharged on the DOS following THA or TKA generally have similar postoperative contacts with the healthcare system when compared with patients not discharged on the DOS.

Total hip arthroplasty (THA) and total knee arthroplasty (TKA) are surgical procedures that have improved continuously perioperatively for many years as a result of implementation of fast-track principles (Husted 2012, Petersen et al. 2019). These changes have led to a reduced length of stay in hospital following THA and TKA while also limiting cost, morbidity, and mortality (Khan et al. 2014, Andreasen et al. 2017, Jørgensen et al. 2017, Burn et al. 2018, Petersen et al. 2020).

The epitome of fast-track surgery is outpatient surgery, where patients are discharged from the hospital on the day of surgery (DOS) to their own homes. This has proven to be beneficial in several ways for selected patients, as these patients spend less time in the hospital while still having similar outcomes when compared with patients not discharged on the DOS with regard to both patient-reported outcome measurements (Husted et al. 2021) and safety (Goyal et al. 2017, Vehmeijer et al. 2018, Gromov et al. 2019). Finally, outpatient THA and TKA come with additional financial benefits (Lovald et al. 2014, Husted et al. 2018, Gibon et al. 2020).

Although an early small study indicated that the reduction in number of hospital days from fast-track did not increase the number of patient contacts with the primary healthcare sector (Andersen et al. 2009), concerns exist that the reduced time patients discharged on the DOS spend in hospital has led to an increased potential burden on other parts of the healthcare system—specifically the primary healthcare system, an increase in readmissions, and/or more contacts with the discharging department as well as other departments (Shah et al. 2019). Therefore, we aimed to investigate whether discharging patients on the DOS after THA and TKA leads to an increased burden on other parts of the healthcare system when compared with patients not discharged on the DOS. This was achieved by comparing readmissions within 3 months, contacts with the discharging department, the surgeon, or other departments—both physical turnouts and by phone, as well as contacts with primary care physicians within 3 weeks.

## Patients and methods

The cohort of patients included in this study is the same as in a previously published study (Husted et al. 2021). That study investigated whether patient-reported outcomes were affected by DDOS after THA and TKA. Patients with an ASA score of 1–2 without sleep apnea requiring treatment, undergoing primary unilateral THA or TKA as 1st or 2nd in the surgical theater between January 2016 and June 2017 were scheduled for same-day discharge as previously described (Gromov et al. 2017, Husted et al. 2021).

All THA and TKA patients had surgery performed by experienced surgeons in a standardized fast-track setup (Husted 2012). Perioperative treatment for the included patients did not differ from standard treatment for all patients operated on during the same period. All patients (also patients not suitable for DDOS who were not included in the study) participated in a preoperative interdisciplinary seminar, covering all aspects of surgery and hospital stay, including information on surgical procedure, postoperative treatment, and discharge criteria. Patients included in the study and eligible for DDOS did not receive any additional information. Spinal anesthesia was used for both THA and TKA patients as well as preoperative single-shot high-dose methylprednisolone (Lunn et al. 2011, 2013). THA and TKA patients were given 2 doses of intravenous tranexamic acid (TXA) preoperatively, and TKA patients received an additional intra-articular dose during surgery. No drains were used for either type of surgery. THAs were performed using a standard posterolateral approach. A standard medial parapatellar approach without the use of a tourniquet was employed for all TKA patients and they all received local infiltration analgesia (LIA) (Andersen and Kehlet 2014). Oral thromboprophylaxis in the form of rivaroxaban was administered 6–8 hours after surgery and continued until discharge.

Postoperatively, patients had a short stay in the postoperative recovery unit after which they were transferred to the orthopedic ward where full weight-bearing mobilization was attempted as quickly as possible. Celecoxib 200 mg/12 hours and paracetamol 1 g/6 hours were used as pain medication for the first postoperative week. Oral morphine 10 mg p.n. was used as a rescue analgesic only. Patients received physiotherapy from the DOS until discharge and patients were referred for public outpatient physiotherapy after discharge for as long as seemed fit.

Specified discharge criteria had to be fulfilled before discharge. These included intraoperative blood loss < 500 mL, pain scores < 3 during rest and < 5 during mobilization (VAS 0–10), spontaneous urination, and successful mobilization. Lastly, all these discharge criteria had to be fulfilled before 8 pm on the DOS, and an adult had to be present with the patient for the first day after discharge.

Information on time of discharge was recorded. Readmissions and complications within 3 months after surgery were recorded using a regional database covering all contacts with the hospital (Gromov et al. 2019). At 3 weeks’ follow-up patients were asked whether or not they had contacted their primary care providers regarding any aspects of the surgery and/or hospital stay, as contacts with primary care are not registered in the regional database. In addition to the readmissions described above, all telephone contacts with the department and/or the surgeon within 3 weeks following surgery were recorded.

### Statistics

Statistical analyses were performed in IBM SPSS Statistics 25 (IBM Corp, Armonk, NY, USA). Normality was tested using the Shapiro–Wilks test after which Pearson’s chi-square test and an independent samples t-test were used to compare data. A statistically significant difference was defined as p < 0.05 when comparing 2 sets of comparable data.

### Ethics, funding, and potential conflicts of interest

No approval from the National Ethics Committee was necessary as this was a non-interventional observational study. The study was approved by the Danish Data Protection Agency (entry no. 20047-58-0015). This work was sponsored by grants from the Lundbeck Foundation and Zimmer-Biomet, which had no influence on any part of the study or on the content of the paper. The authors declare no conflicts of interest.

## Results

From December 2015 through June 2017, 275 patients were scheduled for outpatient THA and TKA. 14 of these were not included in this study because of incomplete data. Among these, 4 were DDOS patients and 10 were nDDOS patients, resulting in 96% data completeness for DDOS and 94% data completeness for nDDOS patients. Therefore, 261 patients remained and were included in this study, consisting of 135 THA patients and 126 TKA patients. 33% (n = 45) of THA patients were discharged on the DOS, whereas 37% (n = 47) of TKA patients managed the same. The remaining patients were all discharged from the ward to their own homes the day after surgery. These results have previously been published (Husted et al. 2021). In order to compare patients accurately, all patients were divided into two groups depending on the type of surgical procedure they underwent: THA or TKA. Furthermore, they were sub-grouped based on whether they were discharged on the DOS or not. The results of this study can be seen in Tables 1 and 2.

**Table 1.** Demographics, numbers, and statistical significance

Factor	DDOS	Not DDOS	p-value
THA patients, n	45	90	
Male, n	33	48	0.03 **^a^**
Mean age (SD)	60 (12)	63 (12)	0.2 ^b^
Mean blood loss, L (SD)	0.31 (0.13)	0.41 (0.28)	0.02 ^b^
TKA patients, n	48	78	
Male, n	20	32	0.9 **^a^**
Mean age (SD)	60 (11)	60 (11)	0.9 **^b^**
Mean blood loss, L (SD)	0.23 (0.10)	0.22 (0.15)	0.8 **^b^**

DDOS = discharge on the day of surgery.

**^a^** Pearson’s chi-square test.

**^b^** Independent samples t-test.

**Table 2.** Readmissions and contacts with the healthcare system. Values are count

Factor	DDOS	Not DDOS	p-value ^a^
THA patients, n	45	90	
Readmissions within 3 months	5	15	0.4
Physical turnouts to discharging			
department within 3 weeks	6	9	0.6
Contact within 3 weeks with			
discharging department by telephone	0	17	0.002
other departments	1	5	0.4
general practitioner	5	15	0.4
TKA patients, n	48	78	
Readmissions within 3 months	5	17	0.1
Physical turnouts to discharging			
department within 3 weeks	17	17	0.1
Contact within 3 weeks with			
discharging department by telephone	14	9	0.01
other departments	5	13	0.3
general practitioner	19	25	0.4

DDOS = discharge on the day of surgery.

**^a^** Pearson’s chi-square test

No statistically significant differences were found between DDOS THA patients and nDDOS THA patients with regard to readmissions within the first 3 months after surgery, physical turnouts to the discharging department, contact with other departments, or with their general practitioners. 11% (n = 5) of DDOS THA patients were readmitted to hospital within 3 months. whereas this was the case for 17% (n = 15) of nDDOS THA patients (p = 0.4). Within the first 3 postoperative weeks, 13% (n = 6) of DDOS THA patients had physical turnouts to the discharging department compared with 10% (n = 9) for nDDOS THA patients (p = 0.6). Only one of the DDOS THA patients (2%) had contact with another department in the first 3 weeks after surgery, whereas this was the case for 6% (n = 5) of nDDOS THA patients (p = 0.4). Among DDOS THA patients, 11% (n = 5) contacted their own general practitioners during the first 3 weeks following surgery, while 17% (n = 15) of nDDOS THA patients did the same (p = 0.4). None of the patients discharged on the DOS following THA had any telephone contact with the discharging department for the first 3 postoperative weeks, whereas 19% (n = 17) of nDDOS THA had telephone contact with the department during this time (p = 0.002).

Among TKA patients, there were no statistically significant differences between DDOS and nDDOS patients regarding readmissions within the first 3 postoperative months, physical turnouts to the discharging department, contact with other departments, or with their general practitioners. 10% (n = 5) of DDOS TKA patients were readmitted to hospital during the first 3 postoperative months and the same was the case for 22% (n = 17) of nDDOS TKA patients (p = 0.1) The discharging department was physically contacted by 35%

(n = 17) of DDOS TKA patients and 22% (n = 17) of nDDOS TKA patients within the first 3 weeks after surgery (p = 0.1). 10% (n = 5) of DDOS TKA patients contacted other departments before 3 postoperative weeks had passed, while 17% (n = 13) of nDDOS TKA patients did the same (p = 0.3). Among TKA DDOS patients 40% (n = 19) had contact with their general practitioners during the first 3 weeks after surgery, whereas this was the case for 32% (n = 25) of nDDOS TKA patients (p = 0.4). A statistically significant difference between DDOS and nDDOS TKA patients was found (p = 0.01) when comparing telephone contact with the discharging department during the first 3 postoperative weeks. 29% (n = 14) of DDOS patients had contacted the discharging department by telephone, whereas this was the case for only 12% (n = 9) of nDDOS patients.

## Discussion

We found that patients discharged on the DOS following THA or TKA did not differ statistically significantly from patients not discharged on the DOS with regard to contacts with the healthcare system. This was the case as readmissions within 3 months were similar between groups. Furthermore, the 2 groups of patients did not differ statistically when comparing physical contact with the discharging department and contact with other departments. Finally, DDOS and nDDOS patients did not differ statistically in terms of contact with general practitioners. These results indicate that discharging patients on the DOS after THA or TKA does not lead to an extra burden on other parts of the healthcare system.

This study is, to our knowledge, one of the first studies to investigate contacts with primary care following outpatient THA and TKA. Previous studies on outpatient arthroplasty have focused on safety, measured as hospital contacts and readmissions, as major complications requiring treatment will most often be readmitted to hospital. We found no difference regarding readmissions when comparing patients discharged on the DOS and patients scheduled for DOS discharge who ended up staying overnight. This is in line with several previous studies that did not find any increased risk of readmissions in selected patients following outpatient THA and TKA (Pollock et al. 2016, Gromov et al. 2019, Xu et al. 2019, Coenders et al. 2020).

Few studies have investigated and compared postoperative contacts between total joint arthroplasty (TJA) outpatients and inpatients. Goyal et al. (2017) compared DDOS and nDDOS THA patients and found a similar number of contacts between the two groups of patients and the office staff. They also found that DDOS and nDDOS patients were similar with regard to healthcare provider visits before their 4-week follow up. Both findings are consistent with our results.

A statistically significant difference was found when comparing the amount of telephone contact with the discharging department among DDOS and nDDOS patients. DDOS THA patients had significantly less telephone contact with the discharging department than nDDOS THA patients, whereas the opposite was the case with regard to TKA patients. Why this was the case is debatable but as we did not register the reasons for telephone contacts, all we can do for now is speculate. One could argue that the shortened length of stay (LOS) in hospital associated with outpatient surgery might lead to less patient education, ultimately resulting in an additional need for more post-discharge contact with the discharging department. However, this would only explain the results of TKA patients in this study. At the same time, the group of patients who are discharged on the DOS might be “fitter” and as a result thereof have fewer complications post-discharge, including readmissions and contact with both the primary and secondary healthcare sector. This may be a confounding factor. Why DDOS THA patients had less telephone contact with the discharging department than nDDOS patients remains inexplicable but pleasing. Not many studies have previously investigated telephone contact after THA and TKA, and, to our knowledge, this has been investigated only by Shah et al. (2019). They compared outpatients with one-night inpatients following TJA and found that the two groups of patients had similar telephone contact with the surgical team post-discharge. They also investigated the subject matters of the phone calls and found that pain, nausea, medication, sleep problems, urination, leg swelling, and physical therapy were the main topics of discussion. These are important findings as they could help facilitate better education of patients both pre- and postoperatively. We did not identify reasons for telephone contacts in our study and therefore cannot compare them directly with the study by Shah et al.

This study has limitations. 1st, a limitation in terms of the nature of our data exists as our data is of a quantitative nature and therefore does not allow for qualitative analyses. 2nd, this study was conducted at a department where outpatient THA and TKA are procedures that have been performed for a long time. Therefore, our results may not be directly transferable to another department where outpatient surgery is not the tradition. However, this could also be seen as a strength, as the department’s routine in outpatient TJA reduces other confounding factors that may affect the results. 3rd, patients included in this study represent a subgroup of patients who were prepared both mentally and physically for outpatient surgery. Therefore, these patients may have been keener on same-day discharge and more tolerant of postoperative complications than other groups of patients. 4th, we only registered patient-reported contacts with primary care, allowing for recall bias. However, we believe it is safe to assume that patients can remember correctly whether they have contacted their primary care physician within the last 3 weeks or not. Finally, it is important to highlight that we investigated selected patients scheduled for outpatient TJA, thus our findings may not be transferable to a wide group of patients.

In conclusion, we found that patients discharged on the DOS following THA or TKA were generally similar with regard to postoperative contacts with the healthcare system when compared with patients not discharged on the DOS; hence these patients do not require extra resources from the discharging department or primary care, even with just a few hours in hospital after surgery. In the future, it would be of great value to investigate the reasons leading to DDOS and nDDOS patients contacting the discharging department by telephone and whether these reasons differ between the two groups of patients. An increased focus on patient education and information could further decrease the number of telephone contacts with the discharging department—especially following TKA. Ergo, this study adds to the mounting evidence that outpatient surgery is a good option for a subgroup of patients.
